# Editorial: Microbial Laccases: Recent Advances and Biotechnological Applications

**DOI:** 10.3389/fbioe.2022.922223

**Published:** 2022-05-25

**Authors:** Abdelmageed M. Othman, Susana Rodriguez-Couto, Tahar Mechichi

**Affiliations:** ^1^ Microbial Chemistry Department, Biotechnology Research Institute, National Research Centre, Giza, Egypt; ^2^ Department of Separation Science, LUT School of Engineering Science, LUT University, Mikkeli, Finland; ^3^ Ecole Nationale d’Ingénieurs de Sfax, University of Sfax, Sfax, Tunisia

**Keywords:** microbial laccases, genetic modifications, overproduction, kinetic modelling, applications

Many sectors are presently investigating enzymatic techniques for creating green chemistry technologies, owing to the drawbacks of physico-chemical processes, rising environmental concerns, regulatory constraints, and expanding scientific understanding. Laccases (E.C. 1.10.3.2) are regarded as one of the most important ligninolytic oxidative enzymes, capable of oxidizing both phenolic and non-phenolic subunits of lignin model compounds as well as highly recalcitrant environmental pollutants, allowing researchers to use them in a variety of biotechnological applications. Laccases are appealing prospects for industrial use due to their broad substrate range and that they only need environmental molecular oxygen to exert their catalytic action, producing water as the only byproduct. In addition, such broad substrate range can be expanded further in the presence of the so-called redox mediators that are small molecular weight organic compounds acting as electron shuttles between the substrate and laccase. Laccases are used in the environmental pollution control, food industry, the paper and pulp industry, the textile industry, synthetic chemistry, cosmetics, soil bioremediation and biodegradation of environmental phenolic pollutants, removal of endocrine disruptors, biosensor and analytical applications (Leynaud Kieffer Curran et al., 2022). Given the numerous applications that laccases can perform; it appears likely that laccases will be one of the most important biotechnological catalysts in the near future.

Laccase-assisted reactions, in particular, are receiving a lot of attention since they are typically eco-friendly and have a lot of application possibilities. Chemically attaching reactive anchor groups to unreactive polymers for subsequent coupling of laccase substrates has broadened application fields as well. Recent chemical or biotechnological approaches to modify enzymes may aid in improving enzyme activity and stability. Numerous strategies for immobilizing laccase have been devised to produce reusable laccase biocatalysts with more stability and oxidizing ability (Daronch et al., 2020; Zhou et al., 2021).

Based on this context, this specific Research Topic has been developed to focus on promising, recent, and unique research developments in the field of microbial laccases. This Research Topic has attracted 31 writers who have contributed to publications. As a result, this Research Topic has provided six peer-reviewed papers to fulfill the needs of the readers, comprising five Original Researches and one Review, all of which covered different elements and corners of this field.

From the point that enzymatic polymerization of lignin may provide a wide range of added-value products while also substituting fossil-based resources. Mayr et al. employed a laccase from the thermophilic fungus *Myceliophthora thermophila* (MtL) as a model substrate to connect a hydrophobicity boosting fluorophenol (FP) molecule onto lignosulfonate (LS). Fluorescence, phenol concentration, viscosity, and molecular weight were all measured throughout the coupling procedure. Full polymerization of LS was accomplished, resulting in the formation of water-insoluble polymers. The incorporation of FP resulted in a rise in water contact angle while a decrease in swelling capacity. The capacity of laccase to facilitate the tweaking of LS characteristics to generate useful polymers is demonstrated in that work.

Kontro et al. used the methylotrophic yeast *Pichia pastoris* to heterologously produce a laccase from the coprophilic basidiomycete *Coprinopsis cinerea* (*Cc*Lcc9). In the presence of redox mediators the *rCc*Lcc9 effectively oxidized veratryl alcohol to veratraldehyde. Furthermore, in the presence of methyl syringate and syringyl nitrile, *rCc*Lcc9 depolymerized biorefinery hardwood lignin. Also, they demonstrated that several added-value aromatic compounds, such as vanillin, vanillic acid, syringaldehyde, syringic acid, and *p*-hydroxybenzoic acid, were formed during the sequential biocatalytic chemical degradation of biorefinery lignin, indicating that *rCc*Lcc9 has a high potential for circular economy and modern biorefinery processes.

For a promising integrated bioprocess for the removal of pesticides in wastewater using crude laccase, Vaithyanathan et al. cultivated *Pleurotus dryinus* on municipal biosolids (BS) as a substrate to manufacture laccase to remove fungicides, herbicides, and insecticides from wastewater. To evaluate the biocatalyst catalytic capability in actual matrices, recovered crude laccase extract was used to remove 29 pesticides (nine fungicides, ten herbicides, and ten insecticides) either separately or as a combination from spiked biologically treated wastewater effluent. Promising findings were achieved using BS-derived crude enzyme extract, which demonstrated enhanced pesticide clearance, which might be attributed to the mediator effect caused by the catalytic transformation of other compounds in the cocktail.

Immobilization has been regarded as a significant strategy for increasing the economic potential of laccase. A critical review was provided by Adamian et al. to discuss new advancements in laccase synthesis, immobilization methodologies, and important support qualities for enzyme immobilization. More precisely, they examined and highlighted the considerable advantages of carbon-based materials in laccase synthesis and immobilization.

With the goal of creating a new and ecologically sustainable wood fiber dyeing technique and producing 3-dimensionally completely colored medium-density fiberboard (MDF). Colella et al. demonstrated the capability of laccase-catalyzed polymerization of chosen precursors to create colors useful in fiberboard manufacture. MDFs were enzymatically colored utilizing a one-step laccase-catalyzed coloring method *in situ*, and the results were compared to commercial MDFs dyed using organic coloring agents. Good color fastness and new hydrophobic qualities where recorded, which allow designers and woodworkers to explore the beauty of textures, as well as the use of simpler and softer processing conditions that minimize harsh chemical use and decrease energy consumption and offer significant advantages over traditional processing methods.

In comparison to the commercial formulation Novozym 51003 with recombinantly generated asco-laccase from *Myceliophthora thermophila*, Euring et al. used *Cc*Lcc5 from colonies of the basidiomycete *Coprinopsis cinerea* as a new basi-laccase in medium-density fiberboard (MDF) manufacturing. *Cc*Lcc5 performed nearly as well as the formulated Novozym 51003 in most evaluated characters. A lignin-laccase-mediator-system (LLMS) bonded MDF was created on a pilot-plant scale using either crude *Cc*Lcc5 or Novozym 51003 at lowered enzyme levels of totally dry wood fiber with lignosulfonate and the redox mediator 2,6-dimethoxyphenol (DMP). Boards made with CcLcc5 performed fairly well as well as Novozym 51003.

## References

[B1] Daronch N. A. Kelbert M. Pereira C. S. de Araújo P. H. H. de Oliveira D. (2020). Elucidating the Choice for a Precise Matrix for Laccase Immobilization: A Review. Chem. Eng. J. 397, 125506. 10.1016/j.cej.2020.125506

[B2] Leynaud Kieffer Curran L. M. C. Pham L. T. M. Sale K. L. Simmons B. A. (2022). Review of Advances in the Development of Laccases for the Valorization of Lignin to Enable the Production of Lignocellulosic Biofuels and Bioproducts. Biotechnol. Adv. 54, 107809. 10.1016/j.biotechadv.2021.107809 34333091

[B3] Zhou W. Zhang W. Cai Y. (2021). Laccase Immobilization for Water Purification: A Comprehensive Review. Chem. Eng. J. 403, 126272. 10.1016/j.cej.2020.126272

